# Promoting Myelination in an *In Vitro* Mouse Model of the Peripheral Nerve System: The Effect of Wine Ingredients

**DOI:** 10.1371/journal.pone.0066079

**Published:** 2013-06-07

**Authors:** Mark Stettner, Kathleen Wolffram, Anne K. Mausberg, Philipp Albrecht, Angelika Derksen, Axel Methner, Thomas Dehmel, Hans-Peter Hartung, Helmut Dietrich, Bernd C. Kieseier

**Affiliations:** 1 Department of Neurology, Medical Faculty, Research Group for Clinical and Experimental Neuroimmunology, Heinrich-Heine-University, Düsseldorf, Germany; 2 Geisenheim Research Center, Department of Wine Analysis and Beverage Technology, Geisenheim, Germany; University Hospital La Paz, Spain

## Abstract

Protective properties of moderate wine consumption against cancers, cardiovascular, metabolic and degenerative diseases have been reported in various clinical studies. Here, we analysed the effect of red wine (RW) and white wine (WW) on myelination using an *in vitro* embryonic co-culture mouse model. The total amount of myelin was found to be significantly increased after RW and WW treatment, while only RW significantly increased the number of internodes. Both types of wine increased rat Schwann cell- (rSC) expression of the NAD+-dependent deacetylase sirtuin-two-homolog 2 (Sirt2), a protein known to be involved in myelination.

Detailed chemical analysis of RW revealed a broad spectrum of anthocyanins, piceids, and phenolics, including resveratrol (RSV). In our assay system RSV in low concentrations induced myelination. Furthermore RSV raised intracellular glutathione concentrations in rSCs and in co-cultures and therefore augmented antioxidant capacity.

We conclude that wine promotes myelination in a rodent *in vitro* model by controlling intracellular metabolism and SC plasticity. During this process, RSV exhibits protective properties; however, the fostering effect on myelinaton during exposure to wine appears to be a complex interaction of various compounds.

## Introduction

The myelin sheath, produced in peripheral nerves by Schwann cells (SCs), is essential for rapid and secure conduction of axonal impulses. In addition, SCs provide trophic support to axons and control regenerative and reparative responses in peripheral nerves [1]. In SCs, various pathways have been identified that are crucially involved in these processes including neuronal cell adhesion, growth factors, and a number of second messenger systems [2], such as the previously identified Sir-two-homolog 2 (Sirt2) gene that has been identified as a putative myelination-associated protein in SCs [3].

Various disorders of the peripheral nervous system can cause marked damage to the myelin sheath that results in relevant clinical impairment of affected patients [4]. Thus, compounds that may promote differentiation of SCs and remyelination are of great interest.

In various clinical studies moderate wine consumption has been suggested to exert beneficial effects in a broad range of disorders [5], [6], [7], [8], [9], [10], [11], [12], [13], [14], [15], [16], [17]. These effects, at least in part, have been linked to polyphenols that can be found specifically in red, but only rarely in white wine [18].

In this study, we analyzed the effect of red and white wine (RW and WW) on developmental myelination using an embryonic tissue mouse model, and aimed to elucidate the molecular mechanism underlying these effects.

## Materials and Methods

### Preparation of dorsal root ganglia

Animal experimentation was approved by local state authorities (Landesamt fuer Natur, Umwelt und Verbraucherschutz Nordrhein-Westfalen) and was in accordance with German European directives. All efforts were made to minimize animal suffering. Dorsal root ganglia (DRGs) were prepared from embryonic C57BL/6 mice (BL6) by opening the cutis and subcutis along the spine and removing the spinal cord not yet completely enclosed by the vertebral column. DRG were collected, centrifuged and resuspended for further treatment [19], [20], [21].

Entire ganglia were plated on 24-well plates (Greiner Bio-One AG, Frickenhausen, Germany). Plates were precoated twice with collagen type I (Becton Dickinson AG, New Jersey, USA) and 0.02 M acetic acid (1∶7), surfaced and dried two times.

DRG cultures were kept in neurobasal medium for 2 days, containing: DMEM (BioWhittacker, Lonza Group AG, Basel, Switzerland), 2 mM L-glutamine (Glut; Gibco, Life Technologies AG, Carlsbad, USA), 10% horse serum (HS; Invitrogen, Carlsbad, USA), 100 U/l penicillin/streptomycin (P/S; Gibco, Life Technologies AG, Carlsbad, USA), 100 ng/ml nerve growth factor (NGF; Sigma-Aldrich Cop., Missouri, USA), and 4 g/l glucose (Sigma-Aldrich Cop., Missouri, USA). Neurobasal medium was subsequently exchanged for myelination media containing minimal essential media (MEM, Invitrogen Corp., Carlsbad, California, USA), 20 µg/ml pituitary extract bovine (PEB, Merck Millipore, Darmstadt, Germany), 50 mg/l L-ascorbic acid (AA, Sigma-Aldrich Corp., Missouri, USA), 0.5 µM forskolin (FKL, Sigma-Aldrich Corp., Missouri, USA), 2 mM L-glutamine (Invitrogen Corp., California, USA), 5% horse HS (Invitrogen, Corp., Carlsbad, California, USA), N2-supplement (N2, Invitrogen Corp., California, USA), 4 g/l glucose (Sigma-Aldrich Cop., Missouri, USA), and 50 ng/ml NGF (Sigma-Aldrich Cop., Missouri, USA). The culture myelination medium was renewed every 3–4 days. Cultures were kept for 28 days *in vitro*, and treated as indicated from the second day after explantation until fixation, followed by staining.

### Pure Schwann cell cultures

Preparation of rat SCs was done using a modified Brockes method [22]. Sciatic nerves were dissected from neonatal (P3) Wistar rats. Cells were plated in DMEM with 10% fetal calf serum (FCS), after digestion with 0.1% collagenase (Worthington, Lakewood, NJ, USA) and 0.25% trypsin (Invitrogen, Carlsbad, CA, USA). To reduce fibroblasts, cultures were treated with two cycles of 10 µM cytosine arabinoside, followed by complement lysis with anti-thymidine 1.1 antibodies with a final purity of more than 95%. Rat SCs were maintained in DMEM Gibco 3185 (Invitrogen, Carlsbad, CA, USA) with 10% FCS, 100 U/ml Penicillin/Streptomycin, 2 mM L-glutamine, and 1 µl/ml FKL.

Cultures were stimulated with RW (Pinot Noir dry 2008, Schloss Reinhartshausen, Rheingau, Germany) at a dilution of 1∶800, equalling 1.25*10^−3^ or 1∶8000, equalling 1.25*10^−4^, as indicated. For WW (Pinot Gris dry 2009, Schloss Reinhartshausen, Rheingau, Germany) identical concentrations were applied, as indicated. RSV (Sigma Aldrich, St. Louis, MO, USA) was dissolved in DMSO, at a stock concentration of 220 mM (50 mg/ml), and used in a concentration of 0.5 mg/l (2.2 µM). Treatment of myelinating cultures with wine or RSV respectively was initiated after removal of neurobasal medium, three days after preparation. Wine was supplemented to myelination medium, concentrations as indicated. Treatment with ethanol and myelination medium without further supplement served as control for wine analysis, DMSO served as control for RSV experiments. Analogously, rSCs or DRG cultures for glutathion (GSH) analysis or immunocytochemistry were treated with wine or RSV for 72 h.

### Sudan staining

Cultures were stained with Sudan black to assess *in vitro* myelination as described before [23]. After washing, cells were fixed for 1 h using 4% paraformaldehyde (PFA, Sigma Aldrich, St. Louis, MO, USA), washed three times with PBS and treated with 0.1% osmium tetroxide for 1 h. After sequential ethanol treatment (25%, 50%, 70% each for 5 min) for 1 h the 0.5% Sudan black solution (Sigma Aldrich, St. Louis, MO, USA), dissolved in 70% ethanol, was added to the cultures, followed by treatment with ethanol at decreasing concentrations (70%, 50%, 25% each for 1 min). Stained myelin of the prepared cell cultures was examined using an upright microscope (Nikon Eclipse TE200, Nikon AG, Tokyo, Japan), analysed by counting the whole number of internodes and correlated to the whole number of neurons within the Sudan-stained culture dishes.

### Food chemistry analysis

Relative density, alcohol, reducing sugars, pH, total acidity (as tartaric acid pH 7) tartaric acid, lactic acid, malic acid, volatile acid and glycerol were determined using Fourier-transform-middle infrared spectroscopy (FTIR) with a Winescan™ 120 (Foss electric, Hilleroed, DK) as described before [24]. Sodium, potassium, calcium, magnesium, iron, copper, and zinc concentrations were determined with high-resolution continuum source AAS (contrAA 300, Analytik Jena). Total polyphenols were analyzed according to classical Folin method as described before [25]. Antioxidant capacity (TEAC) and the ORAC value was analysed as described elsewhere [26], [27]. Individual polyphenols (anthocyanins), resveratrol and other stilbenes, phenolic acids and flavonoids were quantified using HPLC-DAD as described before [28]; for this a Fluofix column 120 E, 2×125 mm, 5 µm (NEOS Company Ltd., Kobe, Japan) was used.

### Immunocytochemistry

For immunocytochemistry the samples were initially washed with PBS and fixed with 4% PFA for 30 min, followed by addition of a PBS-based blocking solution containing 10% natural goat serum (NGS Vector Laboratories Inc., California, USA), 0.1% Triton X-100 (Sigma-Aldrich corp., St. Louis, Missouri, USA), and 0.01% BSA (Carl Roth GmbH, Karlsruhe, Germany) for 1 h at room temperature followed by 15 min 10% natural goat serum (NGS Vector Laboratories Inc., California, USA), 0.01% BSA and 0.5% Triton X-100. Primary antibodies for immunocytochemistry were S100 (1∶100, mouse monoclonal AB, Abcam, Cambridge, UK), SIRT2 (1∶100, rabbit polyclonal AB, Abcam, Cambridge, UK), and SIRT1 (1∶100, rabbit polyclonal AB, Abcam, Cambridge, UK) were used, each diluted in PBS with 10% NGS and 0.1% Triton X-100. Primary antibody incubation overnight was followed by three washing cycles with PBS and incubation with secondary antibody for 1 h at room temperature. The following secondary antibodies were used: anti rabbit IgG, Alexa Fluor 594, PAb goat (1∶400 Invitrogen Corp., California, USA), and anti mouse, respectively, IgG Alexa Fluor 488, PAb goat (1: 400 Invitrogen Corp., California, USA) each diluted in PBS. Samples were embedded with DAPI Vectashield (Vector Laboratories Inc., California, USA) and analyzed with an upright fluorescence microscope (Nikon Eclipse TE200, Nikon AG, Tokyo, Japan and Axioplan 2, Carl Zeiss, Goetingen, Germany). Cells were analysed for optical density, corrected to background fluorescence and correlated to S100 density.

### Green fatty acid assay

The green fatty acid assay or fluorescent fatty acid stain (FFA) allows the detection of myelin structures in living DRG cultures. Therefore C16 fatty acids conjugated with a fluorophore (BODIPY® FL, C_16_, Invitrogen Corp, Carlsbad, California, USA) were added 1∶1000 to the culture media and incubated for 24 h. Due to their low polarity they serve as biological lipid analogue which enables them to be metabolised by lipid enzymes. After a washing step, the marked fatty acids were incorporated into the myelin layer and detected using a fluorescence microscope (Nikon Eclipse TE200, Nikon AG, Tokyo, Japan) at 488 nm. Myelin was quantified using densitometry, analyzing fluorescence of myelinated fibres, normalized to background fluorescence and to the documented surface.

### Glutathione measurement

Total glutathione was measured enzymatically as previously described [29] [30] and normalized to cellular protein measured by the bicinchoninic acid-based Pierce method using a commercially available assay.

### Statistical analysis

Statistical analysis (mean, standard deviation, and P values) was performed with GraphPad Prism software version 4.0 (GraphPad Software, Inc., La Jolla, CA, USA) and calculated using unpaired and paired t-test respectively at 95% confidence interval, one-way ANOVA with Dunnett's test, and applying Mann-Whitney U tests, with P<0.05 considered as statistically significant (P values: *<0.05, **<0.01, ***<0.001). Densitometry for immunocytochemistry or myelin quantification was performed using ImageJ, a public domain image processing program (http://rsb.info.nih.gov/ij/).

## Results

### Wine increases myelination *in vitro*

**Figure 1:**
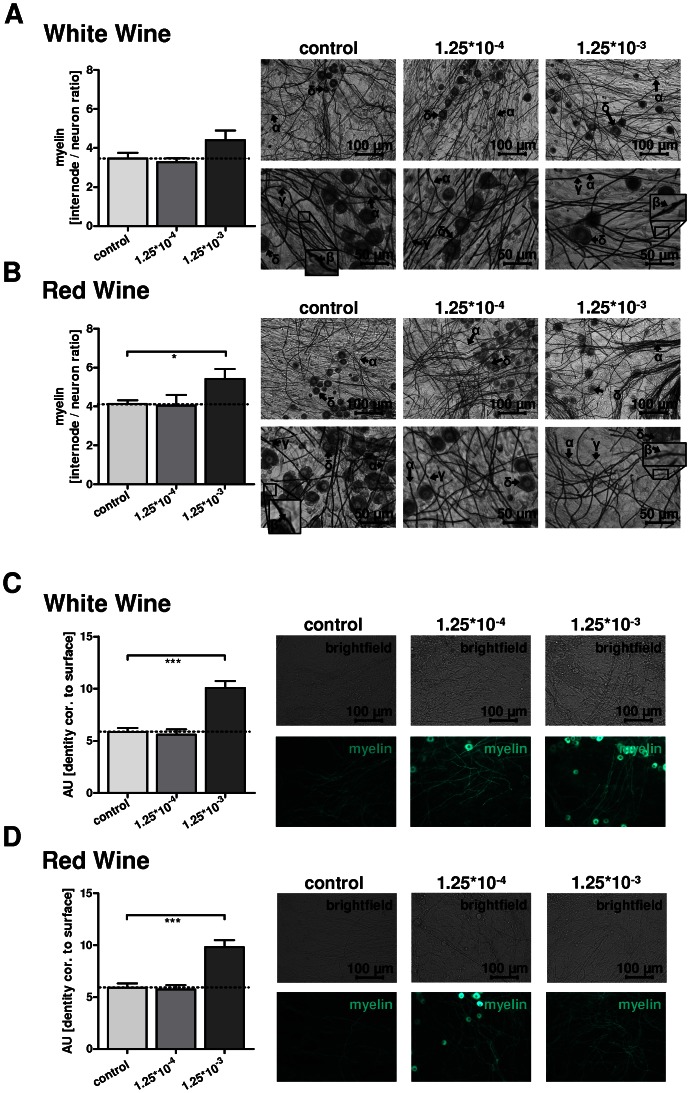
A/B: Treatment of dorsal root ganglia (DRG) cultures with white wine (WW) and red wine (RW, Fig. 1B). Co-cultures were analysed using Sudan Black staining after 28 days *in vitro*. Left: Quantification of myelin synthesis is displayed as total number of internodes correlated to total neuronal number. Right: upright microscopy of Sudan Black stained cultures in the concentrations of 1.25*10^−3^, 1.25*10^−4^ and control stimulation, each displayed in two different magnifications, as indicated. Legends: myelin layer (α) and Schwann cell body (β) are separated from the next internode by the node of Ranvier (γ) and neuron cell bodies (δ). Box indicates a selected area, depicted with higher magnification in order to demonstrate a myelin associated Schwann cell body (β). WW does not increase myelin synthesis significantly but shows in a dilution of 1.25*10^−3^ a slightly increased internode/neuron ratio (p = 0.11). RW in a dilution of 1.25*10^−3^ leads to a significantly increased myelin synthesis 28 days after preparation of explants (p = 0.03). C/D: Quantification of myelin synthesis using fluorescent fatty acids (FFA), which incorporate into the myelin layer. White wine (WW, Fig. 1C) and red wine (RW, Fig. 1D). C: Myelin, quantified by fluorescence of myelinated fibres, normalized to background fluorescence and to the documented surface. Treatment of DRG cultures with WW leads to an increase of myelin density within the cultures for the higher concentration of WW (1.25*10^−3^, p>0.0001), whereas the lower concentration (1.25*10^−4^) did not alter amount of myelin significantly. Right: Representative sectors of the cultures, treated with WW and subsequently with FFA, concentrations as indicated. The top row depicts the bright field images, the corresponding fluorescent images are shown in the bottom row, magnification as indicated. D: Co-culture treatment with RW also leads to a significant increase of total myelin density (p<0.0001) within the cultures for the high RW concentration (1.25*10^−3^), whereas lower concentration did not alter myelin density. Right: As above, representative sector of the cultures, treated with FFA and RW, upper row depicts brightfield images, the bottom row depicts corresponding fluorescent images; concentrations and magnifications as indicated.

Sudan Black staining revealed that treatment with WW resulted in a slight increase of myelin synthesis measured as the number of internodes correlated to neuron number (p = 0.11) when applied at the high concentration of 1.25*10^−3^ (equalling a dilution of 1∶800). The low concentration of 1.25*10^−4^ (equalling a concentration of 1∶8000) did not alter the relative number of internodes significantly (Fig. 1A). The addition of RW in a concentration of 1.25*10^−3^ resulted in a significant increase (p = 0.03) of myelin (Figure 1B). The altered myelin synthesis refers to controls treated with pure myelinating media; controls treated with ethanol (concentrations as measured in wine) did not alter myelin synthesis (data not shown).

Corroborating these results, quantification of cumulative myelin synthesis in the cultures, using FFA staining, which counts tightly-packed as well as less myelinated fibres, revealed a significant increase of myelin after RW (p<0.0001) as well as after WW treatment (p<0.0001) in a concentration of 1.25*10^−3^ (Fig. 1C/D). At low concentrations (1.25*10^−4^) neither RW nor WW significantly altered the total amount of myelin in the cultures, controls as above.

### Chemical analysis of red and white wine

**Figure 2:**
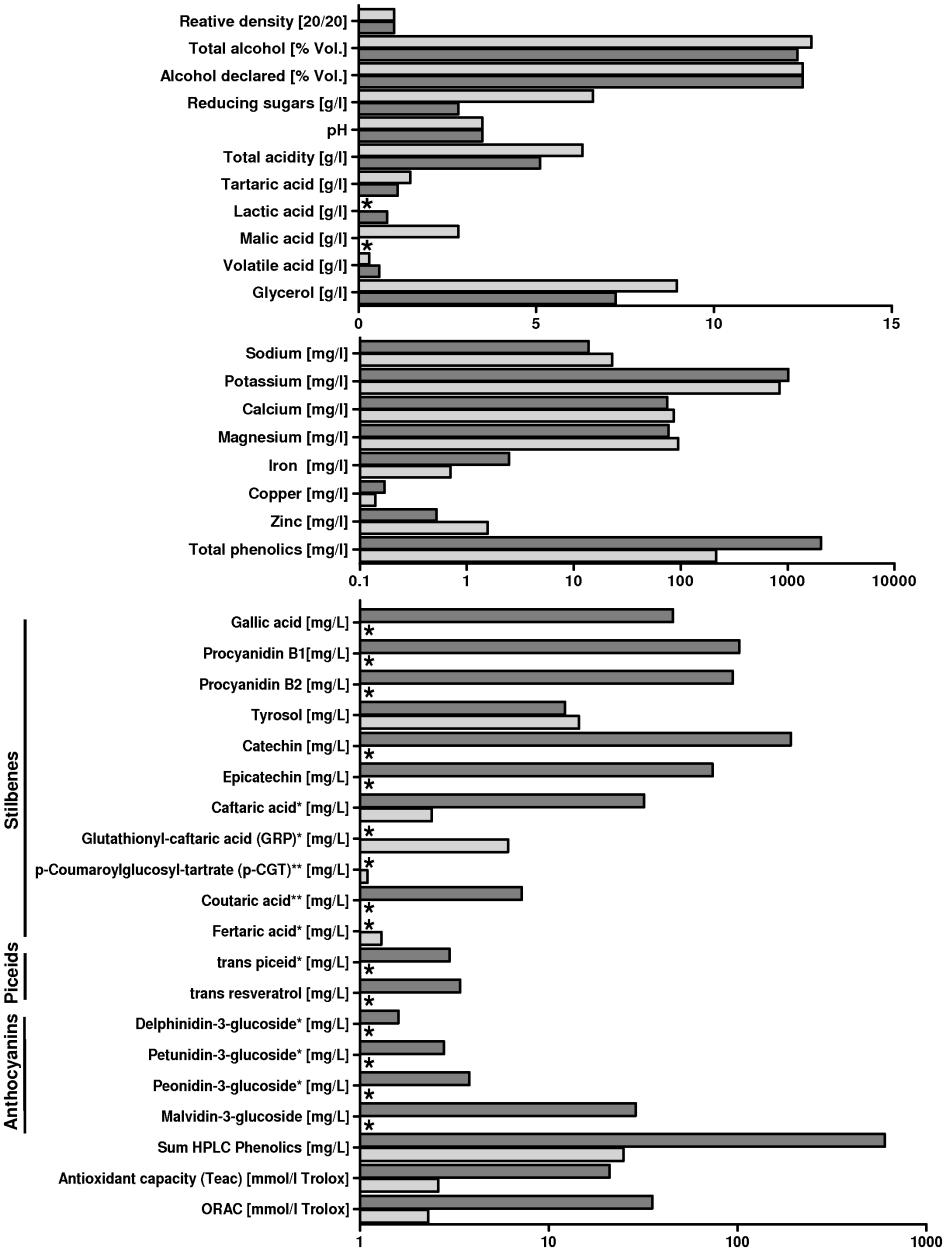
Beverage analysis of red wine (RW; dark-coloured grey bars) and white wine (WW; light-coloured grey bars), which have been used in this study, respectively. (*) indicating that the compound is below limit of detection. The analysed RW compared to the WW contains a broad spectrum of phenolics, stilbenes (piceids and free resveratrol) and anthocyanins. Concerning the basic compounds such as alcohol and ions the two wines are similar.

In order to analyze the individual compounds of the wines used in this study, we performed an organic and inorganic beverage analysis for the major ingredients of wine (Fig. 2 and Tab. 1). As expected, analysis of RW revealed a broad spectrum of phenolics, anthocyanins and stilbenes. Several compounds were not even detectable in the WW, such as procyanidin B1 and B2, catechin, epicatechin, gallic acid, resveratrol (RSV) and anthocyanins. Total phenolic measurement revealed an about tenfold higher concentration in RW compared to WW (2055 mg/l to 215 mg/l), leading to much higher values for TEAC and ORAC in RW. Concerning the basic compounds such as alcohol and ions the two wines were similar.

**Table 1:** Beverage analysis of red wine and white wine, as shown in Fig. 2, displayed numerical.

Wine category		White Wine	Red Wine
Country		Germany	Germany
Region		Rheingau	Rheingau
Vintage		2009	2008
Variety		Pinot Gris dry	Pinot Noir dry
Producer		Schloss Reinhartshausen	Schloss Reinhartshausen
reative density	20/20	0.9956	0.9943
Total alcohol	% Vol	12.74	12.35
Alcohol declared	% Vol	12.5	12.5
reducing sugars	g/L	6.6	2.8
pH		3.48	3.48
Total acidity	g/L	6.30	5.10
Tartaric acid	g/L	1.42	1.1
Lactic acid	g/L	n.n.	0.88
Malic acid	g/L	2.8	n.n.
volatile acid	g/L	0.30	0.58
Glycerol	g/L	8.96	7.24
**AAS**			
Sodium	mg/L	23	13.7
Potassium	mg/L	844	1016
Calcium	mg/L	86	75
Magnesium	mg/L	95	77
Iron	mg/L	0.7	2.49
Copper	mg/L	0.14	0.17
Zinc	mg/L	1.57	0.52
**Phenolics HPLC**			
Gallic acid	mg/L	0.0	45.5
Procyanidin B1	mg/L	0.0	102.3
Procyanidin B2	mg/L	0.0	94.4
Tyrosol	mg/L	14.5	12.2
Catechin	mg/L	0.0	191.6
Epicatechin	mg/L	0.0	73.9
Caftaric acid*	mg/L	2.4	32.0
Glutathionyl-caftaric acid (GRP)*	mg/L	6.1	0
p-Coumaroylglucosyl-tartrate (p-CGT)**	mg/L	1.1	0
Coutaric acid**	mg/L	0.4	7.2
Fertaric acid***	mg/L	1.3	0
*calc.as caffeic acid			
**calc. as coumaric acid			
***calc. as ferulic acid			
**Stilbenes**			
Trans piceid*	mg/L	0	3.4
cis piceid*	mg/L	0	0
Trans resveratrol	mg/L	0	3.3
*calc. as resveratrol			
**Anthocyane**			
Delphinidin-3-glucoside*	mg/L	0	1.6
Cyanidin-3-glucoside*	mg/L	0	0
Petunidin-3-glucoside*	mg/L	0	2.8
Peonidin-3-glucoside*	mg/L	0	3.8
Malvidin-3-glucoside	mg/L	0	28.9
*calc.as mal-3-glucoside			
Sum of HPLC phenolics	mg/L	25.8	602.9
			
Teac mmol/L Trolox	mg/L	2.6	21.0
Orac mmol/L Trolox	mg/L	2.3	35.4
Total phenolics (Folin)	mg/L	215	2055

### Resveratrol promotes myelination in the co-culture model system

For RW a RSV concentration of 3.3 mg/l was measured (Fig. 2, Tab. 1), the corresponding RSV glucoside (trans-piceid) was 3.4 mg/l. The sum of HPLC phenolics in the RW amounted to 602.9 mg/l. Thus the high RW concentration (1.25*10^−3^) contained 0.75 mg/l RSV. Therefore we challenged DRG co-cultures with a comparable RSV concentration (0.5 mg/l RSV), considering RSV as the major polyphenol of the RW used.

**Figure 3:**
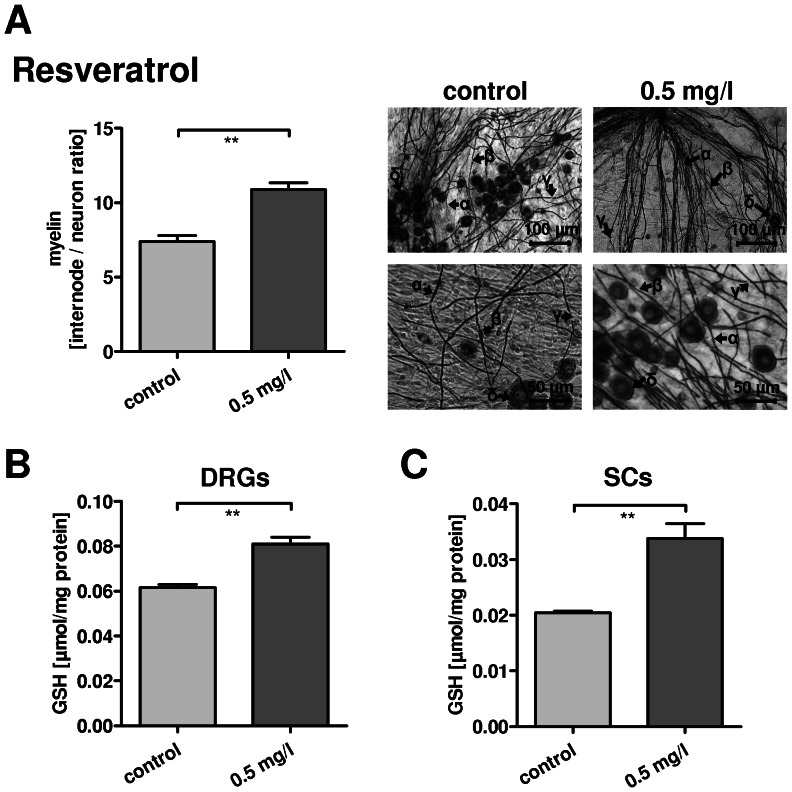
A: Treatment of dorsal root ganglia (DRG) cultures with resveratrol (RSV). *in vitro* using Sudan Black. Left: Quantification of myelin synthesis is graphed as number of internodes correlated to neuronal number in the cultures. Right: Sudan Black staining of co-cultures, treated with 0.5 mg/l RSV, magnification as indicated. Legends: myelin layer (α), Schwann cell body (β), node of Ranvier (γ) and neuron cell bodies (δ). RSV led to an increase of myelin synthesis (p = 0.0062), objectivised by significant increase of internode/neuron ratio. B: Myelinating DRG co-cultures were treated with RSV *in vitro*, concentration as indicated. Glutathion (GSH) was detected in cell lysates as indicator for oxygen stress defence. GSH levels are displayed in µmol/mg of total cellular protein. RSV leads to a significant increase of GSH (p = 0.0022). C: Corroborating to this, treatment of pure SCs led to a significant up-regulation of GSH under 0.5 mg/l RSV stimulation (p = 0.0038).

Cultures were kept for 28 days *in vitro*, and treated as indicated from the third day after explantation until fixation. Myelin expression was analyzed using Sudan Black staining, followed by myelin quantification and morphological analysis (Fig. 3A).

High RSV concentrations (<1 mg/l) lead to a significant decrease of myelin synthesis in the DRG-cultures (data not shown), whereas RSV in a concentration of 0.5 mg/l caused a significant increase of myelin (p = 0.0062, t-test). Myelin morphology appeared unmodified by RSV.

### Resveratrol induces glutathione expression

To assess the potential of RSV to alleviate oxidative stress, GSH concentrations were measured, which are negatively correlated with the antioxidative stress defence. Myelinating DRG co-cultures (Fig. 3B) and SCs (Fig. 3C) were treated with 0.5 mg/l RSV for 72 h. For DRG co-cultures, GSH was detected in cell lysates and showed a significant increase after RSV treatment (p = 0.0022, t-test). Also in pure rat SCs (Fig. 3C) GSH was significantly increased in cell lysates (p = 0.0038, t-test). Treatment of SCs or DRGs with wine did not alter GSH levels to a measurable degree (data not shown).

### Resveratrol induces histone deacetylase SIRT1 expression

**Figure 4:**
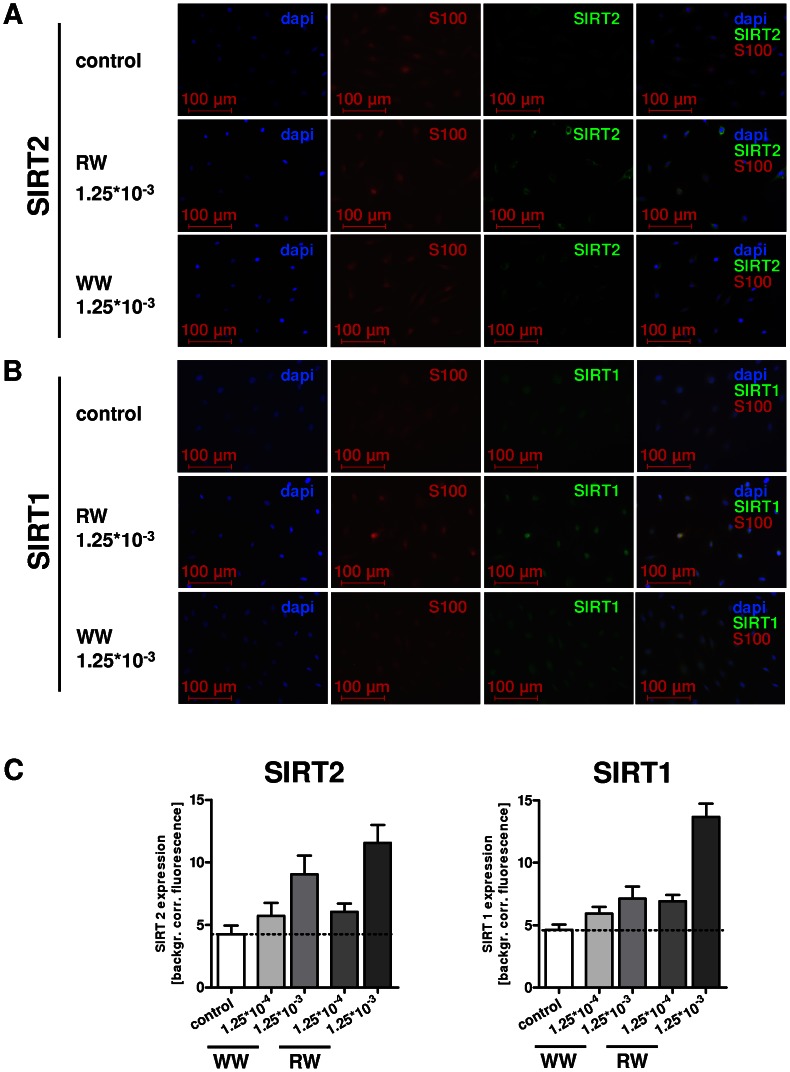
Immunocytochemistry of pure rat Schwann cells (rSCs) for S100 as SC marker, for SIRT2 (A) and for SIRT1 (B) was performed. Staining and magnification as indicated; corresponding merges are shown on the right hand side. Treatment of rSCs with red wine (RW) and white wine (WW) for 72 h, exemplarily shown for a concentration of 1.25*10^−3^. SIRT2, compared to SIRT1 showed a more prominent cytoplasmatic expression in unstimulated SCs. After WW treatment a slightly increased expression and after RW treatment a more prominent increased expression was detected in densitometry analysis (4C). SIRT1 showed faint basic expression, relatively homogeneous for the cytoplasm and for the nucleus. Stimulation with RW or WW led to an increase of expression, emphasising the nucleus and para-nucleus region of the SCs (densitometric analysis Fig. 4C).

**Figure 5:**
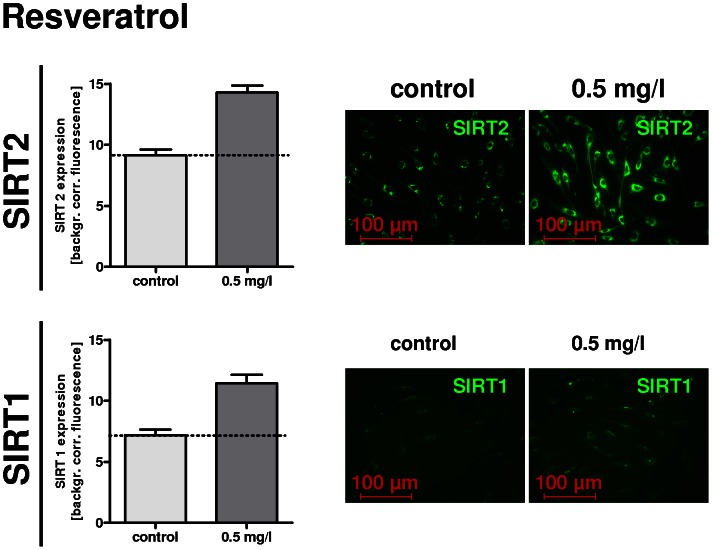
Immunocytochemistry of pure rat Schwann cells (rSCs) for S100, SIRT2 and SIRT1 was performed after resveratrol (RSV) treatment for 72 h. Staining and magnification as indicated. Stimulation of rSCs with RSV led to an increase of SIRT2 as well as of SIRT1 expression in densitometry analysis.

To assess the potential of RW and WW to modify SIRT expression, immunocytochemistry of rat SCs was performed and analyzed densitometrically. Pure rSCs were treated with RW, WW and RSV (concentrations mentioned above) for 72 h. Fig. 4 and Fig. 5 show immunostaining against S100, SIRT2, and SIRT1. SCs displayed expression of SIRT2 and SIRT1, which was enhanced after wine and RSV treatment. Cell number was slightly reduced after RSV and wine treatment.

SIRT2 was detected mainly in the cytoplasm and the expression was slightly increased after treatment with WW or RW in a concentration of 1.25*10^−4^, but clearly elevated under a concentration of 1.25*10^−3^ (Fig. 4A and 4C). The increase was more prominent for RW than for WW (Fig. 4C, immunostaining for 1.25*10^−4^ not shown).

Compared to SIRT2, SIRT1 showed faint basic expression, which was relatively homogeneously distributed in cytoplasm and the nucleus (Fig. 4B, depicted for 1.25*10^−3^, images of 1.25*10^−4^ are not shown, for quantitative analysis see Fig. 4C). Stimulation with RW or WW – in particular RW at a concentration of 1.25*10^−3^ – led to an increased expression, emphasizing the nucleus and para-nucleus region of the cell. Stimulation of rSCs with 0.5 mg/l RSV increased expression of both SIRT1 and SIRT2 as quantified by densitometric analysis (Fig. 5).

## Discussion

We evaluated the effect of a RW and a WW from the same winery using grapes of the burgundy family on peripheral myelin differentiation. In our hands, both RW and WW were found to promote myelination, whereas RW, in concentrations of 1.25×10^−3^ and 1.25×10^−4^, produced more robust effects on the number of internodes as well as the total amount of myelin.

Since wine as a natural compound comprises a variety of organic molecules, we analyzed the major compounds of the wines applied. Not surprisingly, RW contained a broad spectrum of phenolics, anthocyanins and piceids (the glucosides of RSV) as well as free RSV. In regard to basic characteristics, such as ethanol, pH-level, acids, and ions, the two wines used were comparable.

RSV was detectable at moderate levels in the RW but was not measurable in the WW used in the present study. For RSV as major polyphenol neuroprotective properties have been reported [31], [32]. We hypothesized that RSV might account for the superior beneficial effects on myelination when comparing RW vs. WW and indeed noted an increase of myelination after treatment of DRG co-cultures with pure RSV at a concentration of 0.5 mg/l, whereas higher concentrations lead to a decrease of myelin synthesis.

Various favourable effects were observed for RSV or wine treatment in other paradigms and several molecular mechanisms have been proposed in previous reports. In a model of traumatic brain injury, RSV reduced oedema and attenuated pathology by increasing glutathione (GSH) levels [33]. In cerebral and spinal cord ischemia, RSV diminished infarct size [34], [35]. Reduced microglial activation and a decreased neuronal cell death [35], as well as protection of the hippocampal mitochondria via Sirt-UCP2 pathways [34] were discussed as molecular mechanisms. RSV restored depleted GSH [31]. This mode of action was also implicated in an improvement of motor and cognitive function during RSV treatment, noted in the 3-nitropropionic acid-induced animal model of Huntington's disease [32]. In our model of myelination we analysed GSH levels in DRG co-cultures and pure SCs after RSV treatment. RSV increased intracellular GSH concentrations, corroborating the previous observations. Treatment of SCs or DRGs with wine did not alter GSH levels to a measurable degree in our study, an observation which we assume to be caused by the ethanol included in wine. Chronic alcohol consumption can cause damage to the peripheral nerve, causing a wide sprectrum of symptoms such as spontaneous burning pain, hyperalgesia and allodynia [36], [37]. Various mechanisms have been declared responsible for alcohol induced neuropathy including increased vulnerability of the axonal transport system [36], [38], oxidative stress and consecutive damage by free radicals, nutritional deficiency, as well as direct toxic effects of alcohol, or its metabolites e.g. acetaldehyde [36], [39]. Thus, wine abuse *in vivo* causes neuronal and axonal damage, whereas the myelin promoting effects of wine as demonstrated in our *in vitro* system refers to a glial effect.

To further decipher the molecular mechanisms underlying facilitation of myelination, we analyzed SIRT expression. SIRT1 and SIRT2 were detectable by immunocytochemistry in rat SCs, and their expression was modulated by wine; for RW to a greater extent than for WW. Sirtuins comprise a class of nicotinamide adenine dinucleotide (NAD+)-dependent deacetylases (class III HDACs) targeting various pathways to execute diverse biological functions. Sirtuin 1 (SIRT1) is the human homologue of yeast Sirt2. SIRTs are involved in several crucial cellular mechanisms, such as DNA repair and preservation of genomic stability, tumour suppression [40] [41], regulation of p53 function via deacetylation [42], and others [43], [44], [45]. In our experiments both SIRT expression and myelination were enhanced which is in line with recent studies demonstrating an influence of SIRT on structural myelin components during both developmental myelination and remyelination. Most likely this effect is mediated by an impact on essential polarity pathways in SCs during myelin assembly [3]. Thus, our findings support the notion that the observed enhanced myelination after wine treatment may be induced, at least in some parts, by SIRT activation. The slight reduction of pure rat SC numbers under wine or RSV treatment may be caused by cell differentiation, rather than proliferation.

We assume that RSV exhibits protective effects on DRG co-cultures by augmenting antioxidant capacities. The increase in myelination seen under wine treatment, however, appears to be mediated independent from, RSV. This is underlined by the observation that WW, which does not contain RSV, promotes, albeit at a lower magnitude, myelination and by the fact that in higher concentration RSV inverts its myelin promoting properties. Most likely an orchestrated complex interaction of different natural compounds can explain this promotion of myelination. Apart from the direct properties of the flavonoids and other polyphenols, the synergy with e.g. ascorbic acid may also play a molecular role [46]. In particular, the procyanidin B1 and B2 which was detected in high concentrations in the RW used in this study, while not detectable in WW, are candidates for augmenting the effects of ascorbic acid. The involvement of ascorbic acid in PNS myelination is indisputable. Ascorbic acid enables SCs to assemble on the basal lamina, which is required for the differentiation of SCs into a myelinating phenotype. It furthermore promotes the formation of extracellular matrix composed of SC basal lamina and collagen fibrils [47], [48].

The biological activity of RSV and other polyphenols in RW may suggest a beneficial effect of wine consumption. It has been demonstrated, however, that the absorption of polyphenols, such as RSV, when given orally to healthy human subjects reaches only up to 1.9% in serum [49], which may be inadequate to permit biologic activity. Nevertheless, many individuals supplement their diet with RSV-based nutraceuticals [50], with a recommended daily oral intake in a range of 5 mg to 50 mg. Thus, at least theoretically, effective blood level could be reached and the effects noted in our *in vitro* study may be applicable.

In conclusion, RW and also, to a lesser degree WW, promote myelination in the PNS, as demonstrated in an *in vitro* mouse model. The effects of wine on adult human tissue and the relevance of the current results for wine consumers are elusive. The line of evidence needs to be solidified to give a nutrition recommendation concerning the wine selection. While phenolics may exhibit a critical role in the effects observed, further work is required to shed further light on responsible candidates triggering myelination.
