# Correction: Promoting Myelination in an *In Vitro* Mouse Model of the Peripheral Nervous System: The Effect of Wine Ingredients

**DOI:** 10.1371/annotation/a9d91925-7bb9-48a6-b261-40429a319bae

**Published:** 2013-09-20

**Authors:** Mark Stettner, Kathleen Wolffram, Anne K. Mausberg, Philipp Albrecht, Angelika Derksen, Axel Methner, Thomas Dehmel, Hans-Peter Hartung, Helmut Dietrich, Bernd C. Kieseier

The article title was incorrectly given. The correct title is: "Promoting Myelination in an *In Vitro* Mouse Model of the Peripheral Nervous System: The Effect of Wine Ingredients." 

The correct citation is: Stettner M, Wolffram K, Mausberg AK, Albrecht P, Derksen A, et al. (2013) Promoting Myelination in an *In Vitro* Mouse Model of the Peripheral Nervous System: The Effect of Wine Ingredients. PLoS ONE 8(6): e66079. doi:10.1371/journal.pone.0066079. 

